# Model for Interpreting Discordant SARS-CoV-2 Diagnostic Test Results

**DOI:** 10.3201/eid3002.230200

**Published:** 2024-02

**Authors:** Oluwaseun F. Egbelowo, Spencer J. Fox, Graham C. Gibson, Lauren Ancel Meyers

**Affiliations:** The University of Texas at Austin, Austin, Texas, USA (O.F. Egbelowo, L.A. Meyers);; University of Georgia, Athens, Georgia, USA (S.J. Fox);; Los Alamos National Laboratory, Los Alamos, New Mexico, USA (G.C. Gibson);; Santa Fe Institute, Santa Fe, New Mexico, USA (L.A. Meyers)

**Keywords:** COVID-19, respiratory infections, severe acute respiratory syndrome coronavirus 2, SARS-CoV-2, SARS, coronavirus disease, zoonoses, viruses, coronavirus, diagnostic test, rapid antigen test, PCR

## Abstract

We devised a model to interpret discordant SARS-CoV-2 test results. We estimate that, during March 2020–May 2022, a patient in the United States who received a positive rapid antigen test result followed by a negative nucleic acid test result had only a 15.4% (95% CI 0.6%–56.7%) chance of being infected.

During the COVID-19 pandemic, nucleic acid amplification tests (NAATs) and rapid antigen tests (RATs) have been widely used to direct patient care and control transmission (*1*). NAATs, such as reverse transcription PCR, tend to have higher sensitivity and specificity than RATs (*2*) but often are more costly and take much longer to process (*3*,*4*). Thus, RATs increasingly have been used across the United States for at-home symptom-based testing and asymptomatic screening in healthcare, educational, and public event settings (*5*).

During June 2020–April 2022, healthcare providers recommended a confirmatory NAAT after a positive RAT because of high false-positive rates for RATs when community disease prevalence was low (*6*,*7*). When a patient received a negative confirmatory NAAT result, clinicians had to decide which of the results was erroneous and suggest a course of action. 

In this study, we describe a statistical model that can guide the interpretation of discordant test results. The model considers test sensitivity and specificity and estimated community prevalence of the virus. By using community prevalence, the model can estimate the probability that an initial RAT result was a false-positive after a negative confirmatory NAAT result (Appendix).

As a case study, we considered BinaxNOW (Abbott Laboratories, https://www.abbott.com), a test widely used in 2021. BinaxNOW had an estimated test sensitivity of 84.6%; we also considered various NAAT false-negative rates depending on how long after BinaxNOW a NAAT was administered: 68% at 0 days, 37% at 1 day, 24% at 2 days, and 21% at 3 days (*2*). For a patient who received a positive RAT result and then a negative NAAT result, we estimated the probability that the RAT result was erroneous and the patient was not infected (Figure, panel A). That probability was >80% if community prevalence was <200 new weekly COVID-19 cases/100,000 population, the Centers for Disease Control and Prevention (CDC) threshold for low community prevalence (*8*), and generally declined as disease prevalence increased (Figure, panel A). However, a tradeoff exists between NAAT accuracy and speed of diagnosis. For instance, if RAT and NAAT were administered on the same day, the RAT false-positive probability was 89.6% (95% CI 80.5%–100%) when community COVID-19 levels were low according to CDC guidelines. However, if the NAAT was administered 3 days after the RAT, the corresponding probability increased to 96.4% (95% CI 93.0%–100%) (Appendix Table 4). Our confidence in the negative NAAT result peaked when the NAAT was administered 4 days after the RAT (Table; Appendix Figure 1, panel B). Barring other external information (e.g., symptomicity), clinicians can be 89.6% (95% CI 80.5%–100%) confident that the initial RAT result was false-positive when a community is in low risk according to CDC guidelines and 70.5% (95% CI 62.0%–80.5%) confident the same RAT was false-positive when the community is at medium or high risk (Appendix Tables 2–4, Figure 1, panel A).

**Figure:**
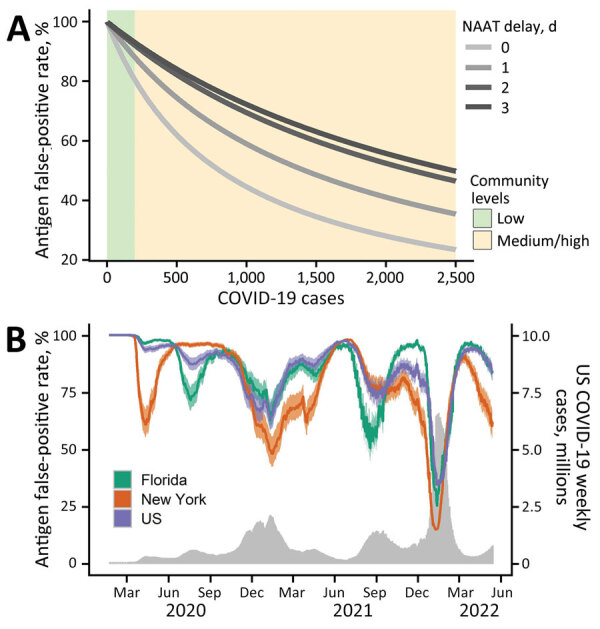
Estimated probability that a positive RAT result is erroneous given a subsequent negative NAAT in a model for interpreting discordant SARS-CoV-2 diagnostic test results. A) Estimated RAT false-positive percentages for levels of community transmission ranging from 0­–2,500 COVID-19 cases per 100,000 population. Green and yellow shading correspond to the Centers for Disease Control and Prevention threshold for low and medium or high community levels (*8*). Line color corresponds to different numbers of days between the initial RAT and confirmatory NAAT, ranging from same day (lightest gray) to 3 days later (black). B) Estimated RAT false-positive percentages for the United States (purple), Florida (green), and New York (orange) during March 2020­–May 2022, assuming the NAAT is administered 1 day after the RAT and that 1 in 4 cases were reported. Shading reflects uncertainty in Centers for Disease Control and Prevention estimated COVID-19 infection underreported, ranging from 1 in 3 to 1 in 5. The gray time series along the bottom indicates the daily 7-day sum of reported COVID-19 cases in the United States. NAAT, nucleic acid amplification test; RAT, rapid antigen test.

**Table:** Probability that a RAT is false-positive in a model for interpreting discordant SARS-CoV-2 diagnostic test results*

No. days between RAT and NAAT	Estimated RAT false-positive rate, % (95% CI)
0	73.4 (49.2–100)
1	82.5 (63.4–100)	
2	88 (73.3–100)	
3	89.2 (75.9–100)	
4	89.6 (76.6–100)	
5	88.8 (75.0–100)	
6	88.4 (74.1–100)	
7	86.7 (71–100)

*The model assumes that a NAAT was negative after a RAT and that NAAT was performed after specified time delay. Estimates assume that the antigen test was performed when patient symptoms first appeared and had a test sensitivity of 84.6% and specificity of 98.54%, which corresponds to the estimated values for BinaxNOW (Abbott Laboratories, https://www.abbott.com) (Appendix Table 1). The NAAT false negative rate for each delay was drawn from a previous study (*3*). NAAT, nucleic acid amplification test; RAT, rapid antigen test.

During May 2020­–May 2022, we estimate that RAT false-positive probability in the United States ranged from 34% (95% CI 29%–41%) to 97.7% (95% CI 97.2%–98.3%), assuming a 25% (95% CI 20%–33%) case reporting rate (Figure, panel B) (*9*). The probability of an erroneous RAT was lowest during the Omicron surge in the winter of 2021–22, when community prevalence was estimated to be highest. At the Omicron peak, we estimate RAT false-positive probabilities of 15% (95% CI 11%–20%) for New York, 25% (95% CI 21%–32%) for Florida, and 34% for (95% CI 29%–41%) the United States (Figure, panel B). The relative trends are similar for other commonly used antigen tests, but the estimated false-positive rates depend on test sensitivities and specificities for each test (Appendix Figures 2, 3).

Rapid and reliable diagnoses of severe infectious diseases is critical for clinical care and infection control. However, the first 2 years of the COVID-19 pandemic revealed enormous barriers to deploying inexpensive, rapid, and accurate tests to combat a newly emerging or rapidly evolving pathogen. We developed this framework during fall 2021 to guide decision-making by patients, physicians, and public health officials in the Austin, Texas, USA metropolitan area. The University of Texas used this model for decision-making regarding when patients might need to visit a clinician. Our framework is limited by the accuracy of the estimates of the RAT and NAAT test sensitivity and specificity and the estimated community disease prevalence, which we drew from transmission estimates from the first 2 years of the pandemic. If community prevalence was higher than we estimated, which could be the case in the early weeks of the pandemic, our model could overestimate the RAT false-positive rate. 

In conclusion, we developed a model to estimate false-positive RAT rates during the COVID-19 pandemic. The model inputs can be readily modified to guide the interpretation of discordant tests as COVID-19 continues to evolve and as RATs become more widely used for other diseases, such as influenza or respiratory syncytial virus (*10*).

Appendix Additional information on a model for interpreting discordant SARS-CoV-2 diagnostic test results.

## References

[1] Wong G, Liu W, Liu Y, Zhou B, Bi Y, Gao GF. MERS, SARS, and Ebola: the role of super-spreaders in infectious disease. Cell Host Microbe. 2015;18:398–401. 10.1016/j.chom.2015.09.013 26468744 PMC7128246

[2] Kucirka LM, Lauer SA, Laeyendecker O, Boon D, Lessler J. Variation in false-negative rate of reverse transcriptase polymerase chain reaction–based SARS-CoV-2 tests by time since exposure. Ann Intern Med. 2020;173:262–7. 10.7326/M20-1495 32422057 PMC7240870

[3] Yang S, Rothman RE. PCR-based diagnostics for infectious diseases: uses, limitations, and future applications in acute-care settings. Lancet Infect Dis. 2004;4:337–48. 10.1016/S1473-3099(04)01044-8 15172342 PMC7106425

[4] Schuit E, Veldhuijzen IK, Venekamp RP, van den Bijllaardt W, Pas SD, Lodder EB, et al. Diagnostic accuracy of rapid antigen tests in asymptomatic and presymptomatic close contacts of individuals with confirmed SARS-CoV-2 infection: cross sectional study. BMJ. 2021;374:n1676. 10.1136/bmj.n1676 34315770 PMC8314145

[5] Filgueiras PS, Corsini CA, Almeida NBF, Assis JV, Pedrosa MLC, de Oliveira AK, et al. COVID-19 rapid antigen test at hospital admission associated to the knowledge of individual risk factors allow overcoming the difficulty of managing suspected patients in hospitals. Fortune J Health Sci. 2022;5:211–31. 10.26502/fjhs.055

[6] Gans JS, Goldfarb A, Agrawal AK, Sennik S, Stein J, Rosella L. False-positive results in rapid antigen tests for SARS-CoV-2. JAMA. 2022;327:485–6. 10.1001/jama.2021.24355 34994775 PMC8742218

[7] Kanji JN, Proctor DT, Stokes W, Berenger BM, Silvius J, Tipples G, et al. Multicenter postimplementation assessment of the positive predictive value of SARS-CoV-2 antigen-based point-of-care tests used for screening of asymptomatic continuing care staff. J Clin Microbiol. 2021;59:e0141121. 10.1128/JCM.01411-21 34288728 PMC8525574

[8] Centers for Disease Control and Prevention; National Center for Immunization and Respiratory Diseases (NCIRD), Division of Viral Diseases. Science brief: indicators for monitoring COVID-19 community levels and making public health recommendations. In: CDC COVID-19 science briefs. Atlanta (GA): Centers for Disease Control and Prevention (US); 2022. 35324136

[9] Centers for Disease Control and Prevention. Estimated COVID-19 burden [cited 2022 May 25]. https://www.cdc.gov/coronavirus/2019-ncov/cases-updates/burden.html

[10] Osterman A, Badell I, Basara E, Stern M, Kriesel F, Eletreby M, et al. Impaired detection of omicron by SARS-CoV-2 rapid antigen tests. Med Microbiol Immunol (Berl). 2022;211:105–17. 10.1007/s00430-022-00730-z 35187580 PMC8858605

