# Supplementary material for: Model for Interpreting Discordant SARS-CoV-2 Diagnostic Test Results
Source: Emerg Infect Dis. 2024 Feb;30(2):388–91. doi: 10.3201/eid3002.230200 (PMC10826750; doi:10.3201/eid3002.230200)
Supplement: Appendix — Additional information on a model for interpreting discordant SARS-CoV-2 diagnostic test results. [file 23-0200-Techapp-s1.pdf]

Article DOI: https://doi.org/10.3201/eid3002.230200

EID cannot ensure accessibility for supplementary materials supplied by authors. Readers who have difficulty accessing supplementary content should contact the authors for assistance.

Model for Interpreting Discordant SARS-CoV-2 Diagnostic Test Results

Appendix

Estimating the Probability of a False-Positive Rapid Antigen Test

Our goal is to estimate the probability that a positive rapid antigen test (RAT) was a false-positive conditional on a subsequent negative nucleic acid amplification test (NAAT) result. Using Bayes' theorem, this is given by the following:

$$P(D-|A=1, N_i=0) = \frac{P(A=1, N_i=0|D-)\cdot P(D-)}{P(A=1, N_i=0)}$$

where *D*– denotes the disease state of the individual (minus and plus indicate uninfected and infected, respectively), A denotes the result of the antigen test and N_t denotes the result of the NAAT when administered *t* days after the antigen test (0 indicates negative and 1 indicates positive). We assume that the antigen and NAATs are independent of one another.

We then estimate the false positive rate as follows:

$$P(D-|A=1, N_i=0) = \frac{P(A=1|D-)\cdot P(N_i=0|D-)\cdot P(D-)}{P(A=1|D-)\cdot P(N_i=0|D-)\cdot P(D-) + P(A=1|D+)\cdot P(N_i=0|D+)\cdot P(D+)}$$

with parameter estimates as described in Appendix Table 1. We assume that $P(D+)$ is equal to the prevalence of SARS-CoV-2 in the community.

Estimating SARS-CoV-2 Community Prevalence

To calculate regional community SARS-CoV-2 prevalence we used daily incident cases provided by the New York Times (*9*). Following a previously described methodology (*10*), we estimated the prevalence for a region, *r*, on the day, *t*, as follows:

$$P_r(D+)(t) = \frac{\sum_{i=t-6}^{t} \frac{C(i)}{\rho}}{N_r}$$

where C(i) is the reported incident cases on day *i*, *P* is the reporting rate, and N_r is the population estimate for the region. We assumed a reporting rate of 25% (20%–33%) during February 2020–June 2022 (*11*) and that infected cases were infectious for 7 days (*12*). We estimated the prevalence for New York, Florida, and the United States as a whole to show how regional variation in epidemic timing impacts the interpretation of discordant test results (Appendix Figure 1, panel B).

References

1. Abbot. BinaxNOW COVID-19 antigen self test [cited 2023 Aug 30]. https://www.fda.gov/media/147254/download

2. AccessBio Inc. on/go COVID-19 antigen self-test: rapid diagnostic test for detection of SARS-CoV-2 antigen [cited 2023 Aug 30]. https://www.code1supply.com/assets/images/08c-EUA210314ongoIFU08-02-2021(2).pdf

3. AccessBio Inc. CareStart COVID-19 antigen test: For use under an Emergency Use Authorization (EUA) only [cited 2023 Sep 2]. https://www.fda.gov/media/142919/download

4. iHealth Labs. iHealth COVID-19 antigen rapid test: healthcare provider instructions for use [cited 2023 Sep 2]. https://www.wellfleet-ma.gov/sites/g/files/vyhlif5166/f/news/ihealth_covid-19_antigen_rapid_test_-_instructions_for_use_healthcare_provider_0.pdf

5. Siemens. CLINITEST rapid COVID-19 antigen self-test: health care provider instructions for use (IFU) [2023 Sep 2]. https://www.fda.gov/media/155175/download

6. Pray IW, Ford L, Cole D, Lee C, Bigouette JP, Abedi GR, et al.; CDC COVID-19 Surge Laboratory Group. Performance of an antigen-based test for asymptomatic and symptomatic SARS-CoV-2 testing at two university campuses—Wisconsin, September–October 2020. MMWR Morb Mortal Wkly Rep. 2021;69:1642–7. PubMed https://doi.org/10.15585/mmwr.mm695152a3

7. Kucirka LM, Lauer SA, Laeyendecker O, Boon D, Lessler J. Variation in false-negative rate of reverse transcriptase polymerase chain reaction–based SARS-CoV-2 tests by time since exposure. Ann Intern Med. 2020;173:262–7. PubMed https://doi.org/10.7326/M20-1495

8. Stadlbauer D, Tan J, Jiang K, Hernandez MM, Fabre S, Amanat F, et al. Repeated cross-sectional sero-monitoring of SARS-CoV-2 in New York City. Nature. 2021;590:146–50. PubMed https://doi.org/10.1038/s41586-020-2912-6

9. New York Times. COVID-19 case data [cited 2022 Aug 1]. https://raw.githubusercontent.com/nytimes/covid-19-data/master/us-counties.csv

10. Chande A, Lee S, Harris M, Nguyen Q, Beckett SJ, Hilley T, et al. Real-time, interactive website for US-county-level COVID-19 event risk assessment. Nat Hum Behav. 2020;4:1313–9. PubMed https://doi.org/10.1038/s41562-020-01000-9

11. Centers for Disease Control and Prevention. Estimated COVID-19 burden [cited 2022 May 25]. https://www.cdc.gov/coronavirus/2019-ncov/cases-updates/burden.html

12. He X, Lau EHY, Wu P, Deng X, Wang J, Hao X, et al. Temporal dynamics in viral shedding and transmissibility of COVID-19. Nat Med. 2020;26:672–5. PubMed https://doi.org/10.1038/s41591-020-0869-5

Appendix Table 1. Assumed model parameters for statistical estimation RAT and NAAT

Parameter	Description	Value								Source
$P(A = 1 \| D–)$	RAT false-positive rate									
BinaxNow		0.0146								(*1*)
On/Go		0.02439								(*2*)
CareStart		0.0068								(*3*)
iHealth		0.019								(*4*)
CLINITEST		0.0071								(*5*)
$P(A = 1 \| D+)$	RAT sensitivity									
BinaxNow		0.846								(*1*)
On/Go		0.87								(*2*)
CareStart		0.9375								(*3*)
iHealth		0.943								(*4*)
CLINITEST		0.854								(*5*)
$P(N_i = 0 \| D–)$	NAAT true-negative rate	0.98								(*6*)
$P(N_i = 0 \| D+)$	NAAT false-negative rate by day	Day, value								(*7*)
		0	1	2	3	4	5	6	7	
		0.68	0.38	0.24	0.21	0.20	0.22	0.23	0.27	
$P(D+)$	Prevalence	0–5%								(*8*)

**D–*, uninfected disease state; *D+*, infected disease state; NAAT, nucleic acid amplification test; *P*(*D+*), SARS-CoV-2 rate in the community; RAT, rapid antigen test.

Appendix Table 2. Thresholds for requiring a clinical visit following a positive antigen test and a negative NAAT confirmatory test*

	% Community prevalence threshold for requiring clinical confirmation	
Desired confidence level, %	1 d between RAT and NAAT	3 d between RAT and NAATs
50	4.3	7.6
65	2.4	4.2
80	1.1	2
90	0.5	0.9
95	0.24	0.46

*Because clinician visits can be burdensome to clinics during a COVID-19 surge and financially costly and disruptive to patients, we recommend requiring a clinician visit when community prevalence exceeds the values provided in the table based on a patient's desired confidence level. For example, if a patient had a positive RAT and negative confirmatory NAAT and wanted to be at least 50% sure that the RAT was a false positive before seeing a clinician, then we would recommend seeing a clinician if the community prevalence was at least 4.3% for a 1-day delay or 7.6% for a 3-day delay between the tests. NAAT, nucleic acid amplification test; RAT, rapid antigen test.

Appendix Table 3. Probability that a positive BinaxNOW RAT is a false-positive given a subsequent negative NAAT, depending on the prevalence of SARS-CoV-2 in the community*

	% Confidence for false-positive RAT after negative NAAT	
Disease prevalence, %	1 d between RAT and NAATs	3 d between RAT and NAAT
0.15	97	98
0.3	94	96
0.5	90	94
1	82	89
2	69	80
3	60	72

*BinaxNOW (Abbott Laboratories, https://www.abbott.com). NAAT, nucleic acid amplification test; RAT, rapid antigen test.

Appendix Table 4. Probability that a positive antigen test is a false-positive after negative NAAT, depending on community prevalence levels*

	% Mean confidence per no. days between RAT and NAAT (95% CI)		
Community levels	0	1	3
Low	89.6 (80.5–100)	93.8 (88.1–100)	96.4 (93.0–100)
Medium or high	70.5 (62.0–80.5)	80.9 (74.5–88.0)	88.4 (84.1–93.0)

*NAAT, nucleic acid amplification test; RAT, rapid antigen test.

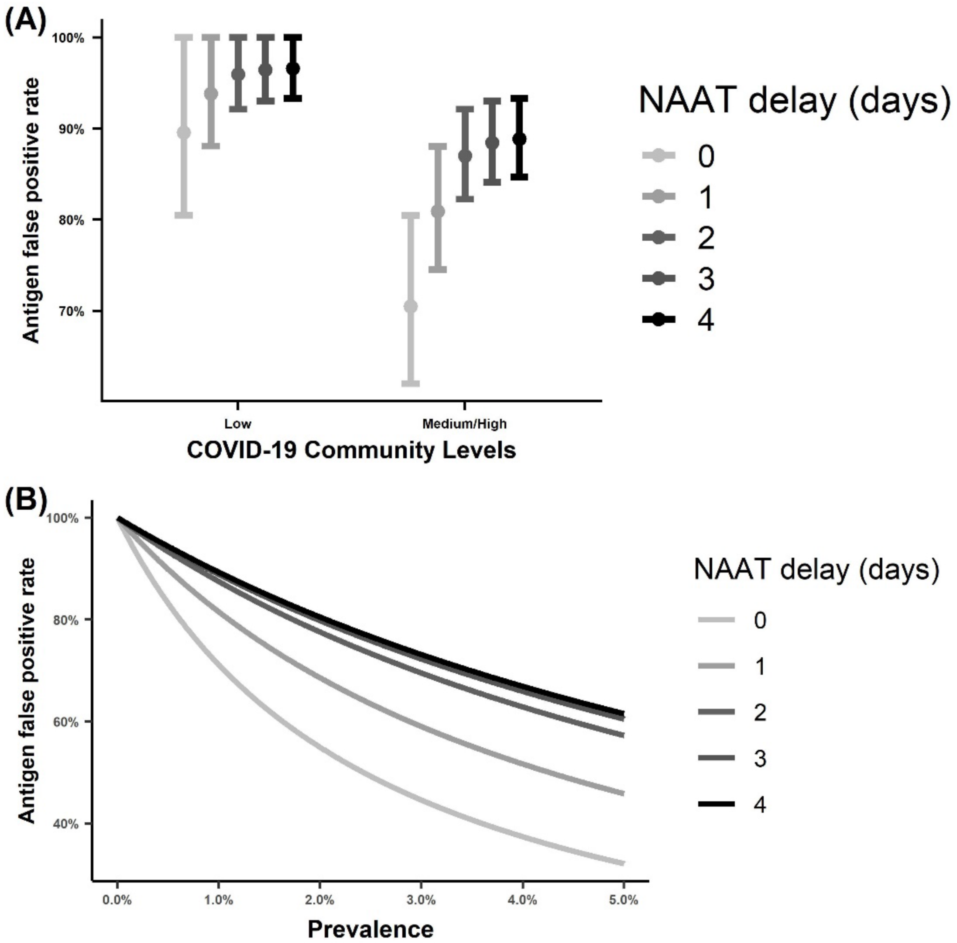

Appendix Figure 1. The probability that a RAT is a false-positive given a subsequent negative NAAT. A) The mean probability that <200 cases (low) and ≥200 cases (medium/high) per 100,000 population are RAT false-positive given a subsequent negative NAAT. Error bars indicate the lowest and highest probability RAT false-positive rates for SARS-CoV-2 community transmission level, assuming that 1 in 4

(95% CI 3–5) infections are reported. B) The probability that a positive RAT is a false-positive given a subsequent negative NAAT, depending on the prevalence of SARS-CoV-2 in the community. Color indicates the number of days between the initial RAT and confirmatory NAAT. NAAT, nucleic acid amplification test; RAT, rapid antigen test.

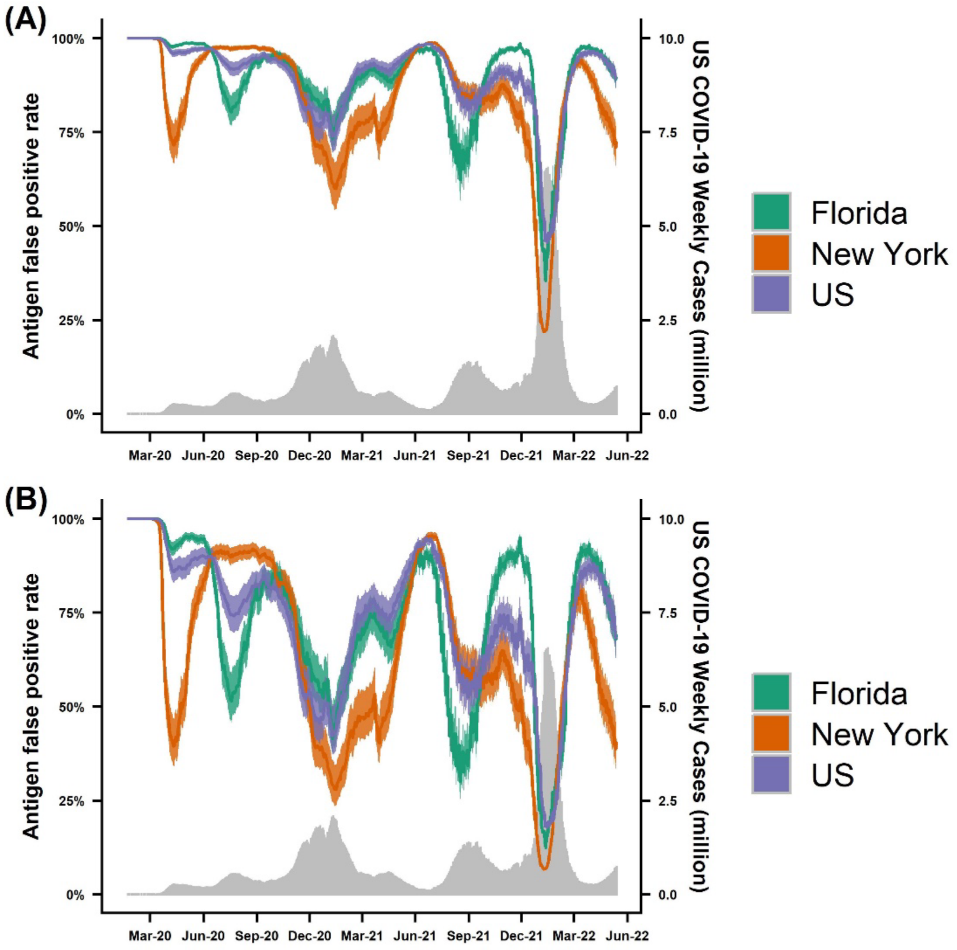

Appendix Figure 2. Estimated RAT false-positive probability rates during March 2020–May 2022, assuming the NAAT is administered 1 day after the RAT and that 1 in 4 infections were reported (*9*). A) On/Go test; B) CareStart test. Colors correspond to the United States (purple), Florida (green), and New York (orange). Shading reflects uncertainty in Centers for Disease Control and Prevention estimated COVID-19 infection underreported, ranging from 1 in 3 to 1 in 5. The gray time series along the bottom indicates the daily 7-day sum of reported COVID-19 cases in the United States. NAAT, nucleic acid amplification test; RAT, rapid antigen test.

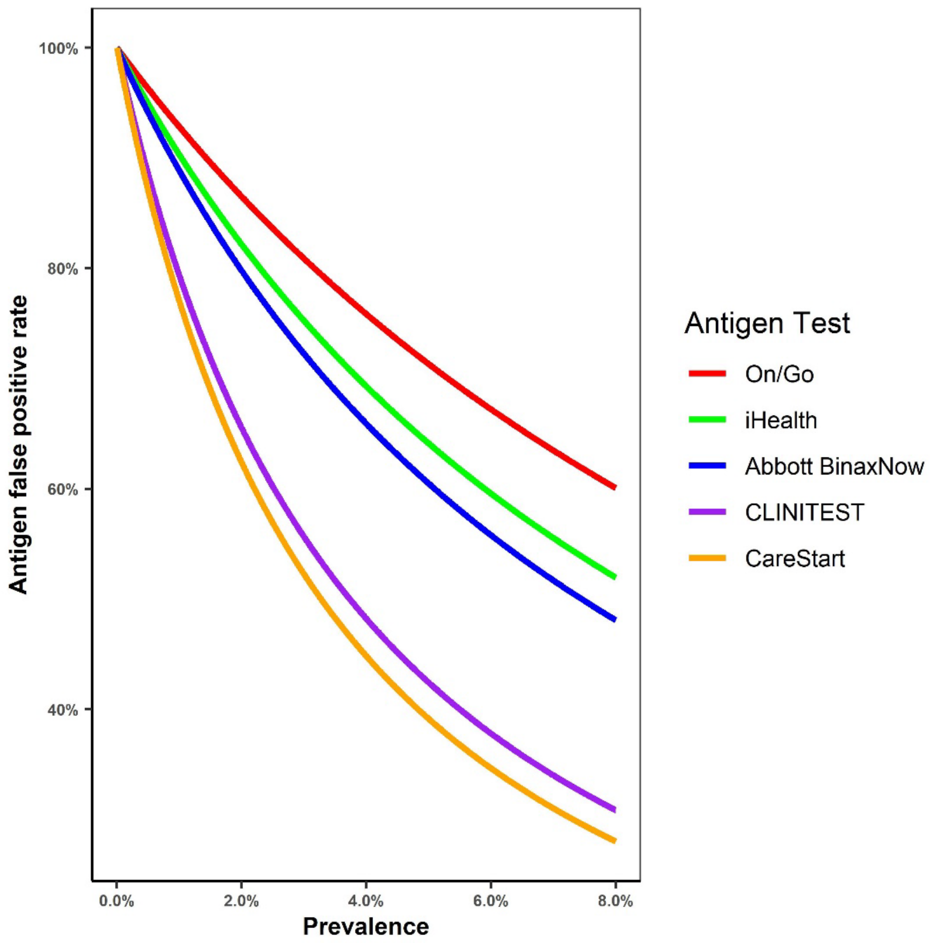

Appendix Figure 3. Estimated rapid antigen test false-positive probability for different community disease prevalences based on the chosen test. Color indicates the specific antigen test, with test sensitivity and specificity as described in Appendix Table 1. BinaxNow (Abbot, https://www.abbott.com); CareStart (Access Bio, https://accessbio.net); CLINITEST (Siemens Healthineers, https://www.siemens-healthineers.com); iHealth (https://www.ihealthlabs.com); On/Go (Access Bio).
